# Experimental Researches into a New Excretory Function of the Liver; Consisting in the Removal of Cholesterine from the Blood, and Its Discharge from the Body in the Form of Stercorine

**Published:** 1862-12

**Authors:** Austin Flint

**Affiliations:** Professor of Physiology and Microscopy in the Bellevue Medical College, New York; &c.


### EXPERIMENTAL RESEARCHES INTO A NEW EXCRETORY FUNCTION OF THE LIVER, &c.

By AUSTIN FLINT, Jr., M.D., Professor of Physiology and Microscopy in the Bellevue Medical College, New York; &c.

(*Continued from page* 697.)

### Stercorine.

The stercorine has already been referred to and delineated in the analyses of various specimens of blood for cholesterine. It was discovered by Boudet, and described by him under the name of seroline, in an article published in the *Annales de Chimie et de Physique*, in 1833, as a principle found in the serum of the blood. Up to the present time, this is the only situation in which it has been found, and here in such an excessively minute quantity that enough has never been obtained for ultimate chemical analysis. With regard to its function nothing whatever has been known. Robin thus speaks of it: "*On ne sait pas comment se forme la seroline, ni quel est son role physiologique.*"*

*Chemical characters.*—This substance, like cholesterine is a non-saponifiable fat. It has never been obtained in sufficient quantity for ultimate chemical analysis; but as in its decomposition it disengages a little ammonia, it is supposed by Verdeil and Marcet to contain nitrogen.† The evidences of this ingredient are very slight, and its existence is doubtful. It is neutral, inodorous, insoluable in water, soluable in ether, very soluable in hot alcohol, but almost insoluable in cold. It is not attacked by the caustic alkalies, even after prolonged boiling. When treated with strong sulphuric acid it strikes a red color, similar to that produced by the sulphuric acid and cholesterine. According to Lehmann it melts at 96° 8′ Fahr., and on the application of strong heat may be distilled without change. Boudet extract ed it from the serum of the blood, by evaporating it, boiling the residue with water, and evaporating again, taking up the residue with boiling alcohol, which deposited the crystals on cooling.

*Form of its crystals.*—Boudet describes the crystals thus obtained as filaments, with varicosities here and there, which gave

* Robin and Verdeil, Chimie Anatomique et Physiologique, tome iii. p. 66.

† Cours de Physiologie fait a la Faculte de Medecine de Paris. Par P. Berard, Professeur de Physiologie, &c., tome iii. p. 118. Paris, 1851.

them a beaded appearance. Lecanu also observed this peculiarity. In the atlas of Robin and Verdeil's *Anatomical Chemistry* we have a beautiful representation of the crystals of seroline from the blood. These observers have not noticed the beaded appearance mentioned by Boudet, but represent the crystals in the form of delicate transparent needles, of variable size, some very small and others quite wide, terminating in a fine pointed extremity, which in some of the wider crystals is bifurcated, or even trifurcated, with the edges of the larger crystals frequently split, as it were, into delicate filaments. The smaller crystals frequently arrange themselves in a fan shape. Robin and Verdeil attribute the beaded appearance mentioned by Boudet and Lecanu to the presence of little globules of fatty matter mixed with the crystals. This seems probable, for we shall see when we examine the process of extraction employed by Boudet, that he probably did not succeed in obtaining it in a pure form. The appearance of these crystals has already been given in some of the diagrams of cholesterine, especially in Figs. 2, 6, 7, and 9. I have been able to follow the process of crystallization in the specimens extracted from the feces from its commencement, and have found that the splitting of the ends and edges of the crystals did not take place at first. The needles which were first found had regular borders and single pointed extremities; but after a few days they split up in the manner described and figured by Robin and Verdeil. (See Figs. 14 and 15.)

*Situations.*—Up to this time the seroline (or stercorine) has only been found in the serum of the blood, and there in but very small quantity, the proportion being, according to the analyses of Becquerel and Rodier, 0.020 to 0.025 of a part per 1,000 parts of blood. They have seen it mount up to 0.060 parts, and descend to a quantity almost inappreciable.*

*Process of extraction.*—In the first observations I made on the blood this substance was observed before the cholesterine. In these observations the blood was dried, pulverized, extracted with ether, the ether evaporated, the residue extracted with boiling alcohol, and then a solution of caustic potash added, which remained on the specimens for a number of days. In all the subsequent analyses of the blood the cholesterine was extracted perfectly pure, and *no stercorine whatever was observed.* The following was the difference in the modes of analysis: In the latter case the solution of potash was not allowed to remain on the specimens more than an hour or two; but was washed

* Traite de Chimie Pathologique Appliquee a la Medecine Pratique. Par M. Alf. Becquerel and M. A. Rodier, p. 62. Paris, 1854.

away, and the residue, which was left on the filter, redissolved in ether. The failure to detect the stercorine in all the latter observations on the blood, which are twenty-four in number, inclines me to the opinion that it does not primarily exist in that fluid; and that when it has appeared in the extract it has been due to a transformation of a portion of the cholesterine. This view seems the more probable, as I have definitely ascertained by observations on the feces, which will be detailed farther on, that the cholesterine is capable of being changed into stercorine, and that this change actually takes place before it is discharged from the body. In my observations on the blood no attempt was made to get rid of this substance, and *though it is soluable in the menstrua which were used to extract the cholesterine, and not destroyed by any of the means that were employed to purify the cholesterine, it never appeared in the extract.* In these exprĭments the study of the stercorine in the blood has not been attempted; and though it is not possible to state at present how the cholesterine was transformed in the first observations, it seems most rational to suppose, in endeavoring to explain its absence in the twenty-four succeeding specimens of blood which were examined, that such a change had taken place.

I am inclined to the opinion, then, though I cannot state it positively, that the element under consideration does not exist in the blood as a proximate principle, but is formed from the cholesterine, in some unexplained way, by the processes which have been used for its extraction.* This transformation does not take place during the extraction of this substance from the feces, because I have in but a single instance been able to extract cholesterine by the processes which are successful in obtaining it in other situations in which it exists, including the meconium.

The stercorine may be extracted from the feces in the following way: The feces are evaporated to dryness, pulverized and treated with ether, which should be allowed to remain from twelve to twenty-four hours, protected from evaporation. The ether is then separated and decolorized by filteration through animal charcoal, fresh ether being added till the original quantity has passed through. It is impossible to decolorize the solution entirely, but it should be made to pass through a very pale amber tinge and perfectly clear. The ether is then evaporated and the residue extracted with boiling alcohol. The alcohol is

* As we have no ultimate analysis of this substance, it is impossible to enter into any chemical speculations with regard to the change from cholesterine, as we may in the instance of the creatine and creatinine, or the urea and carbonate of ammonia.

then evaporated and the residue treated with a solution of caustic potash, at a temperature a little below the boiling point, for from one to two hours. This dissolves all the saponifiable fats, and the solution is then largely diluted with water, thrown on a filter, and washed till the fluid which passes through is perfectly clear and neutral. The filter is then dried at a moderate temperature, and the residue washed out with ether, which is evaporated, extracted with boiling alcohol, and evaporated again. The residue is composed of pure stercorine.* The extract thus obtained is a clear, slightly amber, oily substance, of about the consistence of the ordinary Canada balsam used in microscopic preparations, and in four or five days begins to show the characteristic crystals. These are at first few in number; but soon the entire mass assumes a crystalline form. In a specimen which I have extracted from the feces I have 10.417 grains, consisting, apparently, of nothing but crystals. If the extract be evaporated in a very thin watch-glass it may be examined with the microscope daily, and the process of the formation of the crystals observed. These crystals, after they are fully formed, may be examined satisfactorily with a-half or quarter inch objective.

*History of seroline.*—Very little is to be said with regard to the history of this substance. Boudet first described it in 1833.† Lecanu confirmed these observations in 1837.‡ Since then it has been studied by Becquerel and Rodier, § Chatin and Sandras,|| W. Marcet and Verdeil,¶ Gobly states, in an article published in the *Journal de Chimie Medicale*, that the substance described by Boudet is not an immediate principle but a mixture of several substances, confounding it, however, with cholesterine.** Robin and Verdeil adopt this view, but consider it entirely different from the cholesterine.††

This element, existing, as it does, in large quantities in the

* As this substance is said to be volatilized at a high temperature, it is important, in our examination, to avoid as much as possible the application of heat. Large quantities of it are extracted from the feces after evaporation over an ordinary water bath, but it might be better to evaporate the excrements at a lower temperature.

† Boudet. Loc. cit.

‡ Lecanu. Etudes chim. sur le sang humain, these. Paris, 1837, page 55.

§ Becquerel and Rodier. Recherches relatives a la comp. du sang. (Comptes Rendus. Paris, 144, tome xix. p. 1084.)

|| Chatin et Sandras. Gaz. des hopit., 1849, page 289.

¶ Berard. Loc. cit.

** Gobley. Sur les matieres grasses du sang. (Journal de Chimie Medicale, 1851. Paris, page 577.)

†† Robin et Verdeil. Loc. cit.

fecal matter, must take its place among the important excrementitious substances discharged from the organism, not second in importance even to the urea. It is a curious fact that while the urea was known as an ingredient of the urine long before it could be demonstrated in the blood, taking its name from that fluid, we have the stercorine, an excrement of great importance, discovered in the blood and never till now recognized as an excrement and an ingredient of the feces, taking a name from the serum of the blood, which does not indicate at all its excrementitious properties nor the situation in which it is found in greatest abundance. As seroline has been heretofore a substance of very little prominence, and as it probably does not exist normally in the serum of the blood, and if at all, in insignificant quantity, the application seems a misnomer. We want a name which will indicate its excrementitious properties, and the channel by which it is evacuated, and I have adopted the name STERCORINE* as a more appropriate and more suggestive of its properties, as it is undoubtedly the most important excrement discharged by the anus.

The questions which now arise with regard to this substance open a field of inquiry too extensive, by far, to be thoroughly investigated in the time that could be devoted to this subject, or to be discussed in the limits of this paper. We wish to know the entire history of this product; the quantity discharged in twenty-four hours; variations that may take place with season, age, sex, diet, digestion, &c.; and more especially, the modifications which occur in its discharge in connection with diseased conditions. These points are of great importance, but require a long and laborious series of investigations for their elucidation. What has been done in a measure for the *urea* must be done for the *stercorine*, before we can arrive at a precise idea of its relations to disease. For this purpose a large number of quantitative analyses of healthy feces must be made and compared with similar analyses in different diseases. At present I have only instituted a sufficient number of examinations to substantiate the statements I have made with regard to the formation and discharge of this substance, and added a few examinations of feces in disease which bear upon the same points. I hope at some future time to go more fully into the study of the feces, and contribute something towards the elucidation of some of the questions which naturally arise. In the meantime, I present the following observations on the stercorine as it appears in the feces:—

* From *Stercus*, oris, dung.

*Exp.* 9. Seven and a-half ounces of feces, perfectly normal in appearance, and being the entire quantity passed in the morning at the regular time for an evacuation, were taken from a healthy male, twenty-six years of age. After being evaporated, and pulverized finely in an agate mortar, the residue weighed 2 oz. 57.313 grains. A small quantity was then extracted with alcohol, the solution being of a yellow color, and about six times its volume of ether added. The ether was filtered after standing for fifteen minutes, the filter washed with distilled water, and the solution tested with Pettenkoffer's test for the biliary salts. *None of these salts were present.*

A watery solution was then made of another portion, which was filtered and tested with nitric acid, but failed to show the reactions of the coloring matter of the bile.

The dry residue was then treated with five fluidounces of ether for twenty hours, when it was filtered through animal charcoal, fresh ether being added till the fluid which passed through made five ounces. It came through perfectly clear and of a very light golden tinge. It was then evaporated, leaving a golden yellow fat with a number of whitish resinous masses. It was then extracted with f℥iss of boiling alcohol, which removed everything but a small quantity of bright yellow oil, and filtered while hot. It became turbid on cooling, and was set aside to evaporate. Both the ethereal and alcoholic extracts had a very offensive rancid odor. The residue, after the evaporation of the alcohol, consisted of a considerable quantity of fat of a yellowish color and a consistence like thick turpentine. It was then treated with a solution of caustic potash, kept near 212° for about thirty minutes and allowed to stand for twenty hours. At the end of that time a large quantity of fat floated on the top of the fluid not at all affected by the alkali. It was then largely diluted with distilled water filtered and washed, the filter dried, and the residue redissolved in ether. This ethereal solution was evaporated and the residue extracted with boiling alcohol as before. After the alcohol had evaporated, a small quantity was treated with sulphuric acid, which produced a peculiar red color similar to that produced when the acid was added to a specimen of cholesterine, extracted from the blood and used for purposes of comparison.

Five days after, the specimen was examined with a four-tenth inch objective, and *presented some crystals which looked like seroline;* but it was impossible, on account of the thickness of the glass capsule to apply a sufficiently high power to make this certain. Some long, pale, radiating crystals were observed, composition unknown, but they were not the crystals of excretine described by Marcet.* The specimen was treated again with a solution of caustic potash and kept at nearly the boiling point of water for three and a-quarter hours, most of the fat floating on the top of the fluid in white flakes and yellow drops, but a considerable quantity undergoing saponification, as evidenced by the color of the potash solution. The potash was then removed by filteration, and the residue dissolved in ether and extracted with boiling alcohol as before.

Four days after the evaporation of the alcohol a large number of the characteristic crystals were formed. These did not have the split extremities and edges noted by Robin, but terminated in a single point, and had regular borders; these crystals are represented in Fig. 14.

In a few days the entire mass had assumed a crystalline form, and the crystals then presented split extremities and borders such as are mentioned by Robin. (See Fig 15.)

The quantity of the stercorine was 10.417 grains.

*Exp.* 10. Another analysis was made of the feces from the same individual. During the experiment a large quantity was unfortunately lost, and the examination, therefore, was not quantitative. The presence of stercorine was established.

*Exp.* 11. The feces of the dog from which blood of the carotid and internal jugular on one side had been taken fifteen days

* Marcet, in two papers published in the *Philosophical Transactions*, for 1854 and 1857, describes a new proximate principle in the feces which he calls Excretine. This he obtains in the following way: He first treats the feces with boiling alcohol till nothing can be extracted. A sediment deposits from the alcohol on cooling. The alcoholic solution is acid. Milk of lime is added to the solution, which gives a yellowish brown precipitate, leaving a clear, straw-colored fluid. The precipitate is then collected on a filter, dried, afterwards agitated with ether, and filtered, forming a clear yellow solution. In from one to three days beautiful silky crystals collect in masses, or tufts, adhering to the sides of the vessel, throwing out ramifications in every direction. These, viewed under the microscope, are in the form of acicular, four-sided prisms, and this substance is called by Dr. Marcet, Excretine, and is found nowhere but in the feces. It is soluable in ether and hot alcohol, sparingly so in cold alcohol, and insoluable in hot or cold water. It does not crystallize from an alcoholic solution on cooling, but crystallizes from ether. When suspended in boiling water it fuses into resinous masses, and floats on the top. Its fusing point is 203° to 205° Fahr. It may be boiled for hours with potash without undergoing saponification.

In the article published in 1857, Dr. Marcet gives the composition of the Excretine $C^{78}\ H^{78}\ O^{2}\ S^{1}$.

There is no similarity between the form of the substance described by Marcet and the stercorine. Its high fusing point, 203° to 205° Fahr., and its crystallization from an ethereal solution, also serve to distinguish it from the stercorine, which fuses at 96° 8′., and does not crystallize in an ethereal solution.

before, the animal having entirely recovered, were examined. The analysis was not quantitative. The feces were treated in the way already described, and the presence of stercorine determined.

*Exp.* 12. A specimen of feces voided by a healthy dog, fasting, was examined in the usual way for stercorine. *After the final extract had evaporated, it was examined microscopically, and found to contain, in addition to the stercorine, a considerable quantity of cholesterine, crystallized in beautiful tablets. This is the only examination of feces in which I have found cholesterine.* The proportion of stercorine and cholesterine was as follows:—

| | |
|---|---|
| Quantity of feces, . . . . . . | 137.513 grains. |
| Stercorine with a little cholesterine, . . | 0.216 " |

These examinations of the feces in health show that they invariably contain a non-saponifiable fatty substance known under the name of *seroline*, but which I have called *stercorine*. In but one of these analyses, the last, did I find any cholesterine, though the first were originally undertaken with a view to the extraction of this substance.

The stercorine has never before been detected in the feces, and, as far as my knowledge of its physiological properties is concerned, may be considered a new substance; the discovery of which, in this situation, marks it as one of the most important of the products of destructive assimilation. The next question which arises, then, is with regard to its origin.

*Origin of the stercorine.*—In our study of the chemical properties of this substance, we have already seen that it is one of the non-saponifiable fats, having many characters in common with the cholesterine. It has been described under the name of seroline, as existing in the blood, in very minute quantity, but does not exist in any of the fluids which are poured into the alimentary canal. The cholesterine, however, which it so closely resembles, is one of the constituents of the bile. The fact that the cholesterine is discharged into the small intestine, and not usually found in the evacuations, while stercorine is abundant, would at once point to a possible connection between these two substances. In most cases, in health, cholesterine disappears, and stercorine is found; but in some rare instances, as in the single examination of dogs' feces, Exp. 12, the two substances coexist in the evacuations, the stercorine, in the example just mentioned, in much the greater quantity. The question then arises: Is the cholesterine capable of being converted into stercorine, and does the latter substance originate from a transformation of the cholesterine of the bile? Before we treat of this subject experimentally, let us examine the facts which we already have bearing on this point. No examinations of the feces have ever been made for *stercorine*, but under certain circumstances *cholesterine* has been found discharged by the anus without alteration.

Cholesterine has been detected in the meconium, in the feces of hibernating animals, and occasionally in ordinary feces, though I can find no observation of this kind but the one just recorded.

*Meconium.*—Cholesterine exists in the meconium in considerable quantity, where it may be seen in tablets by a simple microscopic examination, and from which it may be extracted in quantity, and with great facility. The stercorine, or seroline, has never been mentioned as existing in this situation. In the single examination I have made of the meconium I found an abundance of cholesterine, 6.245 parts per 1,000, but no stercorine. There is no difficulty in explaining the origin of the cholesterine in the meconium. Long before any food is taken into the alimentary canal, and before the exclusively digestive fluids are formed, the bile is formed and discharged. It accumulates in the intestine, with other matters constituting the meconium, and is finally evacuated soon after birth. Hence the cholesterine exists in large abundance; but when the digestive fluids are secreted, and food is received into the alimentary canal, the cholesterine is lost and the stercorine makes its appearance.

*Feces of hibernating animals.*—As the excreting function of the liver commences before food is taken into the alimentary canal, so it goes on during the state of hibernation, when the animal takes no food for weeks, or even months. Under these circumstances, the cholesterine is found unchanged in the feces, but it disappears when the animal arouses, and the digestive organs resume their function.

*Normal feces.*—In the normal feces the cholesterine is generally absent; but in Exp. 12, it was found in small quantity, mixed with stercorine. This animal had been certainly twenty-four hours, and probably forty-eight hours, without food. The feces were of normal color and consistence.

These facts seem to show that before digestion commences, as in the fœtus, and when it is suspended, as in hibernating animals and in Exp. 12, cholesterine passes through the alimentary canal unchanged. But as soon as digestion commences, the cholesterine is lost in the feces, and its place is supplied by stercorine. It seems almost certain, then, that in its passage down the alimentary canal, the cholesterine of the bile is acted upon by some of the digestive fluids and changed into stercorine. This change seems to be incident to the digestive act; for before digestion commences, or when it is suspended, the cholesterine passes through unchanged. A conclusive observation would be to cut off the bile from the intestines, and consequently the cholesterine, and notice the effect upon the production of stercorine. In a case of jaundice from duodenitis (which will be more minutely detailed in the section on the pathological relations of cholesterine,) the necessary conditions for this observation seemed to be fulfilled. The patient suffered from intense jaundice, dependent upon obstruction of the common bile duct from duodenitis. The feces were clay-colored. After a time, the patient was relieved of the jaundice, and the feces regained their natural color. While the feces were decolorized, and when the icterus was most marked, it is probable that the bile was entirely cut off from the alimentary canal. This condition was relieved, however, when the feces regained their color, and the icterus disappeared. For the purpose of ascertaining the effect of the obstruction to the flow of bile on the stercorine in the feces, and of the re-establishment of the flow, the stools were examined chemically during the jaundice, and after the patient had recovered.

*Analysis of decolorized feces.*—The quantity of feces examined was 941.4 grains. After evaporation, extraction with ether, and extraction of the residue left after the evaporation of the ether, with hot alcohol, the fat, which was very abundant, was entirely saponified by boiling for fifteen minutes with a solution of caustic potash, *showing that neither cholesterine nor stercorine was present.*

*Analysis of feces from the same patient after they had become normal in color.*—This analysis was made nineteen days after the preceding one. The quantity of feces was small. The specimen was treated in the usual way, showing stercorine in the following proportions:—

| | |
|---|---|
| Quantity of feces, . . . . . . . | 502. grains. |
| " stercorine, . . . . . . | 0.34 " |

Taken in connection with the facts which have already been cited with regard to the discharge of cholesterine by the anus, when digestion is not going on, this observation establishes the origin of the stercorine. *It is produced by a transformation, connected with the digestive act, of the cholesterine of the bile.* When the cholesterine does not find its way into the alimentary canal, as was the case in the first analysis of feces, stercorine is not found in the dejections; when the discharge is re-established, the stercorine reappears.

*Comparison of the daily quantity of stercorine discharged, with the quantity of cholesterine produced by the liver.*—The quantity of stercorine which I extracted from the regular daily feces of an adult healthy male, was 10.417 grains. As there is no cholesterine found in the dejections, this should represent the entire quantity of cholesterine excreted in the twenty-four hours. A comparison of this quantity with the estimated quantity of cholesterine discharged in the day, shows this to be the case.

| | |
|---|---|
| Quantity of bile in the twenty-four hours, (Dalton) . | 16.940 grains.* |
| " " cholesterine at 0.618 pts. per 1,000, (A. Flint, Jr.) | 10.469† " |
| " " stercorine discharged, (*Id.*) . . . . . | 10.417 " |
| Difference, . . | .052 |

This insignificant difference of .052 of a grain, at once proves the correctness of the estimate of the daily quantity of bile excreted, the accuracy of the estimate of the proportion of cholesterine in the bile, and of the quantitative analysis for the stercorine; and made, as the three observations were, without the slightest reference to each other, adds the final link to the chain of evidence in support of the view that *the cholesterine, in its passage down the alimentary canal, is converted into stercorine, in which form it is discharged in the feces.*

The history of the cholesterine thus resolves itself:—

1. *Cholesterine is an effete material, produced by the destructive assimilation of nervous matters, and absorbed by the blood.*

2. *It is separated from the blood in its passage through the liver, enters into the composition of the bile, giving this fluid its excrementitious character.*

3. *It is poured with the bile into the upper part of the small intestine, when the process of digestion induces a change into stercorine; in which form it is discharged by the feces.*

4. *Stercorine, the great excrementitious element of the feces, is one of the most important excrements produced by the waste of the system.*

Pathological Relations of Cholesterine.—With the limited data we have on the subject of the variations in the quantity of cholesterine in health and disease, it is impossible to do more than merely to open the great subject of its pathological rela-

* Dalton's Treatise on Human Physiology, 2d epition, page 171.
† See table, page 760.

tions. To a certain extent, all questions in physiology have for an end the elucidation of points in pathology. The practical physician, who may be the reader of this article, will naturally inquire if the more definite views which we are now enabled to hold with regard to the function of the bile be of any use to him in the study and treatment of disease. It is certain that no addition to our knowledge of the functions of the healthy body is without its bearings on disease, immediate or remote. What may seem to be simply a matter of interest to the pure physiologist, without apparently any practical bearing, is sure at some time to be so connected, perhaps, with other advances, as to be useful to the practitioner. But the pathological relations of an important excretion do not have a practical interest so remote, especially when this function is connected with the liver. Almost from time immemorial, a large number of diseases have been referred to derangement of the liver, and in their treatment, it has been thought of immense importance to promote the secretion of the bile. A certain class of remedies supposed to regulate the function of the bile, has been constantly employed by physicians. At the present day, these ideas have fallen somewhat into disrepute; for the enlightened physician is now accustomed to base his pathological views upon a certain amount of definite knowledge, and it has been found that both the physiology and the pathology of the bile have been very little understood. The older practitioners had, as we have now, a certain class of cases characterized by a general *malaise*, and having indefinite symptoms which were attributed to "biliousness," in which they were in the habit of employing the cholagogues, with mercury at the head, with undoubted success. It is true that, as our knowledge of disease becomes more accurate, the conditions which were supposed to indicate "biliousness," have become referred to other disorders; but no great advance has been made in pathology of the liver, and there are yet many conditions which may be successfully—though empirically—treated, the true character of which is unknown. It is on this obscure subject that it is hoped the preceding physiological investigations will throw some light. To repeat an illustration made use of before, a knowledge of the functions of cholesterine, and its history in the healthy organism, should contribute as much to the pathology of diseases dependent on derangement of this function, and has the development of the functions of the urea for diseases now known to be dependent or uremia.

CHOLESTEREMIA.

In common with other excrementitious substances, which invariably exist in the blood in health, if the function of the eliminating organ be interfered with, accumulation takes place in the blood. This fact has already been incidentally referred to in treating of the properties of the cholesterine which allied it to effete substances. It takes place with the urea; but cases of uremic poisoning occurred, and patients died in uremic coma, long before the cause of it was understood. It is the same with the bile. Ordinary cases of jaundice, which have been called, by Piorry, cholemia, are not of a dangerous character; but there are cases in which jaundice, though less marked as regards color, is a very different condition. Here we have evidently the operation of some poison in the blood, and coma and death from its effects on the brain, follow as in retention of the urea. Pathologists inquire why there is this difference in the severity of cases of icterus? Chemists have analyzed the blood in the hope of explaining it by the presence of the glyco-cholates and tauro-cholates of soda in the grave cases, regarding them as the only important constituents of the bile. But their failure to detect these substances has left the question still unanswered.

*In simple cases of jaundice we have a resorption of the coloring matter of the bile from the excretory passages.*

*In grave cases of jaundice, which almost invariably terminate fatally, we have a retention of the cholesterine in the blood, or cholesteremia.*

I have been forced to make use of cases of disease exclusively in studying this condition, for no one has yet been able, in the larger animals, to extirpate the liver, as we do the kidneys, notice the symptoms of poisoning, and demonstrate the accumulation of cholesterine in the blood. Nor have I yet been able, on account of the insolubility of the cholesterine, to make experiments by injecting it into the circulation. I had, however, an opportunity of making an examination of the blood of a patient in the last stages of cirrhosis of the liver, accompanied with jaundice, and compare it with an examination of the blood of a patient suffering from simple icterus. Both of these patients had decoloration of the feces; but in the first, the icterus was a grave symptom, accompanying the last stages of the disorganization of the liver; while in the latter, it was simply dependent on duodenitis, the prognosis was favorable, and verified by the result. As icterus accompanying jaundice is of very infrequent occurrence, I deem myself very fortunate in having an opportunity of comparing the two cases.

CASE I. *Jaundice dependent on obstruction from duodenitis.*—Mary Bishop, æt. 42, native of Ireland, widow, occupation servant, was admitted into the Blackwell's Island Hospital, June 12, 1862, with the following symptoms: Slight febrile movement, with severe pain over the duodenum; the surface of the body was highly icterosed; the stools were clay-colored; urine high-colored, but not examined for bile; lungs and heart normal; appetite rather poor; no ascites. The icterus had existed since about May 23, 1862. The patient was confined to the bed.

Dr. Flint, the attending physician, pronounced it a case of icterus dependent on duodenitis.

*Treatment.*—Laxitives daily, with good diet and a moderate amount of stimulus.

*June* 21. A small quantity of blood was drawn from the arm for examination, and *June* 23, the feces were collected for the same purpose.

*June* 27. The patient remains about the same.

*July* 11. All pains and tenderness over the duodenum have disappeared. She has steadily improved since the last record. The stools have been natural for several days. Though confined to the bed most of the time, she is able to sit up two or three hours daily. The jaundice has been gradually diminishing, and three or four days ago it had entirely disappeared, and is now absent.

*July* 12. Another specimen of the feces, which was of normal appearance, was taken for examination.

*Analysis of the blood for cholesterine.*—The blood was examined about sixteen hours after it was taken from the arm. It had entirely separated into serum and clot. The serum was of a bright yellow color—more markedly bilious than in the succeeding case. It was evaporated, pulverized, and a quantitative analysis for cholesterine made, with the following results:—

| | |
|---|---|
| Quantity of blood, . . . . . . . | 212.428 grains. |
| Quantity of cholesterine, . . . . . | 0.108 " |
| Proportion of cholesterine per 1,000 pts. of blood, | 0.508 " |

CASE II. *Jaundice with cirrhosis.*—Ann Thompson, æt. 39, native of Ireland, occupation servant, was admitted into the Blackwell's Island Hospital June 16, 1862, and gave the following history:—

Three months ago she contracted a severe cold, which was accompanied with swelling of the left hand and of both legs, continuing for eight or nine weeks. At the end of that time, she noticed that the abdomen was increasing in size. She was then very weak, the urine was scanty, bowels regular up to the time when she entered the hospital. She denied having been in the habit of drinking spirit, but acknowledged that she drank beer.

*June* 18. The surface of the body was very much icterosed, the color being very marked under the tongue and in the conjunctiva; the abdomen was full of fluid; pulse 90, small and weak; bowels loose, and the dejections clay-colorcd; the urine highly tinged with bile and copious; appetite very poor. She was tapped, and about eight quarts of clear, straw-colored serum were evacuated. The patient was confined to the bed.

Dr. Flint, the attending physician, diagnosticated cirrhosis.

The treatment consisted of sustaining measures, with stimulants, and the tinct. ferri muriat.

*June* 21. A small quantity of blood was taken from the arm for examination, and *June* 23, a specimen of the feces was obtained for the same purpose.

The patient died June 27. There were no convulsions, and she was sensible, though in a state of stupor, up to twenty minutes before the fatal termination. The stupor existed three or four days before death. Two days before, she complained of double vision. The icterus was excessive up to the time of her death.

*Autopsy.*—The abdomen contained about twelve quarts of fluid. The liver was examined; its weight was 3lbs. 12½ oz. It was very light-colored, and had something of the "hob nail" appearance; presenting, in short, the gross characters of cirrhosis. The gall-bladder was very much contracted, and contained only about two drachms of bile. Microscopic examination of the organ showed the liver-cells shrunken. The fibrous substance was increassd in quantity, and there were present a large number of rather angular globules of fat.

*Analysis of the blood for cholesterine.*—The blood was examined about sixteen hours after it was drawn. It had fully separated into serum and clot, and the serum was of a greenish, yellow color. The whole, serum and clot, was then evaporated, pulverized, and a quantitative analysis made for cholesterine in the manner already indicated, with the following results:—

| | |
|---|---|
| Quantity of blood, . . . . . . . | 50.776 grains. |
| Quantity of cholesterine, . . . . . | 0.093 " |
| Proportion of cholesterine per 1,000 pts. . . | 1.850 " |

The following table gives a comparison of these results with those obtained in the analyses of the three specimens of healthy blood from the arm, which were examined at the same time, and all five specimens subjected to identical processes.

*Table of Quantity of Cholesterine in Healthy Blood, Blood from Simple Jaundice, and Jaundice with Cirrhosis.*

| Healthy Blood. | Cholesterine per 1,000 pts. | Blood of Jaundice. | Cholesterine per 1,000 pts. |
|---|---|---|---|
| Male, æt. 35, . . . . | 0.445 | Case I. Simple Jaundice, | 0.508 |
| " " 22, . . . . | 0.658 | " II. Jaundice with Cirrhosis, . . | 1.850 |
| " " 24, . . . . | 0.751 | | |

| | | |
|---|---|---|
| Case I. Simple Jaundice. | Percentage of increase over minimum of healthy blood, . . | 14.157 |
| | Decrease below maximum, . | 32.357 |
| Case II. Jaundice with Cirrhosis. | Increase over minimum, . | 315.730 |
| | Increase over maximum, . | 146.338 |

The results of the examination of the blood in these cases of disease are very striking and instructive. We have already seen that the variation in health are very considerable. In the three analyses here noted, the maximum was 0.751, and the minimum 0.445 pts. per 1,000. The conditions which regulate this variation it has not yet been possible to study; but we know enough with regard to it to see that in the examination of blood in disease, the cholesterine must mount considerably above the maximum, or fall much below the minimum, to be considered beyond the limits of health. But in the second specimen of jaundiced blood, the variation from the limits of health is so considerable as to enable us to draw very important physiological and pathological conclusions.

In the first place, what is the bearing of these observations on the *physiology* of cholesterine? As before remarked, no one has been able to remove the liver from a living animal, and notice the effect upon the quantity of cholesterine in the blood.* This experiment, if it were possible, and if it showed that the cholesterine increased in quantity, and killed the animal, would be positive proof that it was an excrementitious substance, and that it was removed by the liver. But while the experimental physiologist contributes much to the information of the pathologist by artifically producing abnormal conditions, pathology furnishes a multitude of useful experiments of Nature, if we

* Muller, Kunde, and Moleschott have succeeded in extirpating the liver from frogs, and keeping them alive for two or three days—Moleschott preserving them for several weeks. These observations were made with reference to the accumulation of the biliary salts, and of the bile pigment in the blood, their attention not having been directed to cholesterine. A series of experiments on frogs was commenced by the writer, but they promised to be so prolonged that they were deferred.

may so term them, which are invaluable to the physiologist. In the present instance, cases of disease of the liver present a condition which we are not at present able to imitate by experiments on the lower animals. Disorganizing disease of the liver must interfere with its excretory function, as Bright's disease does with the elimination of urea; and if cholesterine be an excrementitious substance to be removed by the liver, when the liver is seriously affected with structual disease, we will have an accumulation of it in the blood. *This, if fully established, is positive proof of the character of cholesterine and the function of the liver connected with its elimination.*

What do we learn then by a comparison of the blood in Case II. of jaundice dependent on cirrhosis, with healthy blood and a study of the history of the case?

The cholesterine, in this instance, is enormously increased in quantity, 315.730 per cent over the minimum, and 146.338 per cent over the maximum. The case, as far as symptoms are concerned, was of a very grave character. The patient not only suffered from an accumulation of fluid, but there was evidently a poison in the system. The patient died after three or four days of stupor, and, on *post mortem* examination, the liver was found disorganized. There was a deficient secretion of bile, and had been for a long time, for the gall-bladder was very much contracted, and the stools were clay-colored. In short the patient died of *cholesteremia;* and the fact that this condition can exist is a proof of the excrementitious function of the cholesterine, as uremia, as a toxemic condition, presupposes that urea is an excrement.

Physiologically this case fulfils the essential condition of excrementitious substances; namely, accumulation in the blood when the eliminating functions of the excretory organs are interfered with. The liver became so disorganized that its functions were seriously embarrassed, and the quantity of cholesterine in the blood increased to an enormous extent.

The *pathological* deductions from the facts which have been elicited by the examination of the blood in these cases seem to me of great importance. The literature of diseases connected with disorders of the liver is full of theories, more or less plausible, to explain certain conditions which have long been established by clinical observation. We have cases of simple jaundice which are not dangerous to life, and sometimes, though the icterus be excessive, run a certain course without even interfering with the ordinary avocations of the patient. Again, we have jaundice, which is invariably fatal. That disease described by Frerichs under the name of acute atrophy of the liver, and called by some acute jaundice, is one of the most serious diseases of which we have any knowledge. The existence of this great difference has led clinical observers to attribute the mild cases to simple resorption of the *coloring* matter of the bile, and the severe cases to a retention or resorption of some of its more important constituents; especially as it has also been observed that the symptoms which characterize the latter condition occasionally occur in structural diseases of the liver without any discoloration of the skin. But the pathology of this disease has been entirely unknown. It has been thought that in such cases some of the elements of the bile should exist in the blood. Frerichs says: "In the same way that urea accumulates in large quantity in the blood in granular degeneration of the kidneys, so ought the biliary acids and bile-pigment to accumulate in the blood in cases of granular liver. Repeated observations have proved that this is not the case."* Experiments on animals have been followed with like results. The frogs that were kept alive by Moleschott for weeks after the removal of the liver did not present a trace of any of the biliary salts or pigment in any part of the system, showing that these matters are manufactured in the liver. This obscurity, which leads to all sorts of theories with regard to liver pathology, must exist as long as our knowledge of the physiology of the bile is so indefinite. If observers had looked for cholesterine —a substance which pre-exists in the blood and is separated by the liver—instead of the biliary salts—which do not pre-exist in the blood, are manufactured in the liver, as these experiments tended to show, and are peculiar to the bile—they would have met with different results. The very fact that the biliary salts are peculiar to the bile, and found in no other fluid, should have led them to disregard this substance in their analyses of the blood, because this stamps it as a secretion and distinguishes it from the excretions; they should have looked for some substance which exists in the blood as well as the bile, an indispensable condition of an excretion, and this substance is the cholesterine.

Understanding, as we now do, the physiological relations of the cholesterine, we may divide jaundice into two varieties: simple *Icterus*, or yellowness, and a condition which I have called *Cholesteremia.* We may have, also, the latter condition unaccompanied by discoloration of the skin.

* A Clinical Treatise on the Diseases of the Liver, by Dr. Fried. Theod. Frerichs, Prof. of Clinical Medicine, &c., Berlin. Translated by Charles Murchison, M.D. The New Sydenham Society, London, 1860, vol. i. p. 83.

*Simple icterus.*—In simple icterus we have a resorption of the coloring matter from the biliary passages. As it has been proven that the coloring matter of the bile appears first in the liver, when it exists in the blood, it is not due to accumulation, but to resorption. In these cases the resorption is generally dependent on obstruction from some cause, by means of which the bile is confined. The patient suffers only from the disease which causes the obstruction, and from the derangement of digestion which is due to the absence of bile in the intestinal canal. In those cases, in which we have no organic lesion of the liver, there is no danger of absorption of the cholesterine. We have a condition which is analogous to retention of urine. The patient suffers simply from retention of bile in the excretory passages, and cholesteremia is no more to be expected from obstruction of the bile duct without structural changes of the liver, than uremia is to be looked for in vesical retention of urine without organic change in the kidneys. Excrements are not reabsorbed, though they may be retained in the blood.

The quantity of cholesterine in the blood is not necessarily increased in simple icterus, for the liver is still performing its function of eliminating it, and when once separated from the blood it is not taken up again. The analysis of the blood in Case I. indicated a proportion of 0.508 parts per 1,000, which is within the limits of health; a little below the mean, probably on account of the somewhat enfeebled condition of the patient.

The feces may or may not be decolorized, this depending on the extent of the obstruction to the passage of bile into the intestine. The obstruction to the flow of bile is frequently removed before the system has time to remove the coloration of the skin, and the feces become normal while the patient is icterosed. In some instances there is no change in the appearance of the dejections during the course of the disease. When the dejections are entirely decolorized there is an absence of the *stercorine*, into which the cholesterine is transformed before it is discharged, with an abnormal quantity of fat which has passed through undigested.* This element reappears in the feces when the flow of bile is re-established and they assume their normal color.

The two following analyses were made of the feces in Case

* This fact, which has often been remarked, seems to indicate that the bile is actively concerned in the digestion of the fats. I have noticed that dogs with biliary fistulæ, though with a ravenous appetite, refuse to eat fat meat. This disinclination to eat fat has been noticed in cases of jaundice with decoloration of the feces.

I.; one, when they were entirely decolorized, and the patient was very much icterosed, and the other, when she had recovered, and the dejections had assumed their natural appearance.

*Feces of Case I. Jaundice dependent on duodenitis First analysis.*—The feces were clay-colored, and apparently destitute of bile. They weighed 941.4 grains. They were evaporated to dryness without difficulty, pulverized, digested with f℥iij of ether for twenty hours, filtered through animal charcoal, evaporated and extracted with hot alcohol. The fatty residue after evaporation of the alcohol was very abundant.

The residue was then treated with a solution of caustic potash and exposed to a gentle heat. In fifteen minutes it became entirely saponified, forming a clear homogeneous soap with no residue, showing the entire absence of stercorine. The soap was boiled down and moulded into a cake, which I preserved and which weighs 34 grains. The following are the results of the examination:—

| | |
|---|---|
| Quantity of feces, . . . . . . . . | 941.4 grains. |
| " fat, . . . . . . . . | 39.124 " |
| Percentage of fat, . . . . . . . . | 4.144 |

No stercorine or other non-saponifiable fats.

*Feces nineteen days after. Second analysis.*—At that time the patient had entirely recovered from the jaundice, and the feces had regained their natural appearance. A small specimen was taken for chemical examination, which was done, employing the process already described for the extraction of stercorine, with the following results:—

| | |
|---|---|
| Quantity of feces, . . . . . . . . | 503. grains. |
| " stercorine, . . . . . . . | 0.340 " |

*Cholesteremia with icterus.*—In jaundice complicated with blood poisoning we have a very different state of things as regards gravity of symptoms and prognosis. This occurs in acute jaundice, or when it accompanies, and is dependent upon, structural change in the liver, as the jaundice of cirrhosis. The difference in the pathology of these cases, compared with those of simple icterus, has long been recognized; but, as before remarked, analysis of the blood has failed to throw any light on the subject, because chemists directed their attention exclusively to the biliary salts. Frerichs says:—

"I have myself repeatedly examined jaundiced blood, which has been obtained by venesection, or still more frequently, from the heart or venæ cavæ of the dead body, for the biliary acids, and their immediate derivatives; and more recently I have had it examined by my assistant, Dr. Valentin, but always with negative results. No substance could be found in the alcoholic extract of the blood which yielded any indication, by Pettenkoffer's test for the biliary acids, whether this alcoholic extract was treated directly with sulphuric acid and sugar, or whether, in order to get rid of foreign substances, a watery extract of it was first prepared. This coincides with the experience of most of the older observers."*

In cases of blood poisoning by retention and accumulation of elements of the bile in the blood, we have, as the important pathological condition, the great increase in the quantity of cholesterine. The fact of accumulation of this substance in the blood in certain cases of icterus, has been noticed by Becquerel and Rodier; but they do not connect it with structural change in the liver, and do not explain its physiological or pathological importance. The fact of its accumulation in the blood is strong evidence of its excrementitious function, but this does not appear to have attracted the attention of the observers just mentioned. The following is one of the cases in which increase of the cholesterine was observed by them, the only one in which they allude to its significance, and here merely to state their inability to explain it:—

" The second case is nearly similar, excepting the phlegmasia, which did not exist. It relates to a boy, nineteen years of age, a *limonadier*, affected for some time with a bilious diarrhœa, with fever, and icterus, recently developed and very marked. There existed in the blood of this patient a slight diminution of the globules (136); albumen in normal quantity (71.4); likewise fibrin (2.3); fatty matters sufficiently abundant; seroline in an imponderable quantity; cholesterine excessively abundant (0.798); soaps abundant (2.032) *To what cause must we attribute this great quantity of cholesterine? How and why is it concentrated in the blood in spite of the biliary flux? This is what it is difficult to decide.*"†

The acquaintance we now have with the physiology of cholesterine removes the difficulty in the explanation of this fact. In Case II. we had the rare complication of jaundice with cirrhosis, the symptoms evidently pointing to poisoning by retention of some noxious element in the blood. Examination of the blood in this case showed that the cholesterine existed in the proportion of 1.850 parts per 1,000; the minimum of healthy blood being 0.445 and the maximum 0.751 parts. Taking this case as an example, we have cholesteremia with jaundice presenting symptoms which characterize the retention of bile in the blood, which are already well known, and were established long before we were able to say what element was retained. Cases in which jaundice exists with cholesteremia are so different from cases of ordinary jaundice that there is no difficulty in making the discrimination by symptoms. When jaundice exists

* Frerichs, op. cit., page 95.

† Translated from Becquerel and Rodier, op. cit., page 210. The Italics are my own.

with cirrhosis, it is probable that we always have cholesteremia. In acute jaundice the symptoms, especially those referable to the nervous system, are so marked that the gravity of the case is easily recognized. I have no doubt but in such cases the cholesterine is immensely increased in the blood, though on account of their rarity I had not had an opportunity of determining this by analysis. Icterus with cholesteremia and simple icterus are as distinct from each other as possible. The only feature they have in common is the discoloration of the skin. The simple icterus, which is comparatively harmless, is not liable to run into the more severe variety, which cannot occur without structural change in the liver; while the grave variety occurs when we have evidence of organic lesion of the liver, or presents symptoms from the first which indicate its serious character. The one has no more constitutional danger than exists in a simple case of spasmodic retention of urine, while the other has characters as grave as those which accompany uremic poisoning from disorganization of the kidney.

In these cases the feces may show a very marked deficiency of bile, but this is due to the deficient, but not arrested, secretion of this fluid, while the clay-colored stools in simple icterus are dependent on the want of discharge of the bile into the intestine. While in the latter instance, as in Case I., we would expect to find an absence of stercorine in the dejections, in the former we would expect to find it, though in greatly diminished quantity. Further examination of the feces in cases of structural disease of the liver will be of immense advantage as indicating, by the quantity of stercorine found, the extent to which the eliminative function of the liver is interfered with.

The following analysis was made of the feces in Case II. of jaundice dependent on cirrhosis.

*Feces of Case II. Jaundice dependent on cirrhosis.*—The feces were clay-colored, though it was not quite as marked as in the Case I. of simple jaundice.

The specimen was evaporated with great difficulty. It came down to a black glutinous mass which could not be pulverized. It was treated twice with alcohol, the alcohol evaporated, and once with ether. After the ether had evaporated it was pulverized and analyzed for stercorine, with the following results:—

| | |
|---|---|
| Quantity of feces, . . . . . . . . . | 272.1 grains. |
| Stercorine, . . . . . . . . . . | 0.077 " |

In this analysis we have a very great diminution of the stercorine in the feces, the normal quantity in the daily passages, according to the single examination I have made, being 10.417

grains. The specimen was the ordinary amount passed daily. It showed that the cholesterine was still eliminated, though not with sufficient activity to prevent its accumulation in the system. This was further evidenced by the *post mortem* examination, when the gall-bladder was found contracted, but containing a small quantity of bile.

Examination of the blood and feces of the patient suffering under cholesteremia with jaundice, thus leads to the following conclusions:—

1. *The cholesterine is enormously increased in the blood, showing that the structural change in the liver has interfered with its removal from that fluid.*

2. *The stercorine is correspondingly diminished in the feces, showing that the cholesterine is not discharged in normal quantity into the alimentary canal.*

*Cholesteremia without icterus.*—In a practical point of view, this condition becomes one which it is very important to be able to recognize; but it is here that we feel most the necessity of more extended investigations than it has been possible to make. I have been enabled only to open the subject by the analysis of one or two specimens of blood taken from patients who had organic change in the liver, but no jaundice. One of the most familiar of these affections of the liver consists in those changes of structure which are included in the term cirrhosis. It is very unusual to find this associated with jaundice, and we have already seen how serious this symptom is. Frerichs describes a condition which he calls *acholia*, denoting suppression of the functions of the liver. This is the same condition which I have called *cholesteremia*, which expresses the element of the bile which produces the toxic effects, the action of which was unknown to Frerichs, while the term acholia expresses retention of bile without giving us any idea of the active morbific agent. Further investigation will, undoubtedly, establish more fully what the analyses I have made thus far seem to show, namely, that in what Frerichs calls acholia without jaundice, we have the cholesteremic condition we have seen to exist in acholia with jaundice. The following quotation from Frerichs' admirable treatise on the liver, gives an idea of one of the conditions in which we have acholia (or cholesteremia) with or without jaundice.*

"Cases have repeatedly occurred to me, in which individuals who for a long period have suffered from cirrhosis of the liver, have suddenly presented a series of symptoms which are foreign to that disease. They have become

* Frerichs, op. cit., page 241.

unconscious, and have been afterwards seized with noisy delirium, from which they passed to deep coma, and in this state have died. In one case there was spasmodic contraction of the muscles of the left side of the face. In most cases, slight jaundice made its appearance at the same time, and in one instance there were petechiæ. Upon *post mortem* examination, not the slightest lesion could be detected in the brain, neither were there indications of any acute disease which could account for the derangement of the cerebral functions. The liver, in all cases, presented cirrhotic degeneration in a marked degree, and the glandular cells were for the most part loaded with fat; large quantities of leucine separating from it; the bile ducts contained only a small quantity of pale bile."

In certain cases of organic lesion of the liver, and probably in all cases accompanied by the grave symptoms mentioned by Frerichs, we have cholesteremia; but this character does not exist in all cases where the liver is affected, any more than uremia exists in all cases of structural disease of the kidney. Nature not only provides organs which are sufficient for the removal of effete matters from the blood, but provides for conditions in which the function of these organs may be partly interrupted, and yet the excretion go on, a part taking on the function of the whole. One of the kidneys may be removed, and yet the other, increased in sized it is true, is capable of performing the function of both. The kidneys may be partially disorganized, and yet the sound portion be sufficient for the depurative function, and urea will not accumulate in the blood. So it is with the liver. We see patients with partial disentegration of this organ, as in some cases of cirrhosis, suffering apparently but little inconvenience from the disease, and presenting none of the symptoms of cholesteremia. But when the liver is extensively affected, so much so that it cannot separate the cholesterine effectually from the blood, we have the condition of cholesteremia. I have made an analysis of the blood of two patients affected with cirrhosis, who presented this contrast as regards the symptoms of cholesteremia. In one of them, Case III., there was considerable constitutional disturbance; and in the other, Case IV., the patient was about, and suffered no great inconvenience, though he had been tapped for ascites about thirty times.

Case III. *Cirrhosis with ascites, and considerable affection of the general health.*—Mary Perkins, æt. 23, native of Ireland, prostitute, has been a spirit-drinker for about seven years, about the first of May, 1862, noticed an enlargement of the abdomen, which was accompanied with pain over the region of the liver, when she took to the bed. She states that at that time the stools were dark green. Fluid continued to accumulate in the abdomen, and was drawn off in the hospital (Blackwell's

Island), June 25. About six quarts of a clear, straw-colored serum were removed, but a little was left in the abdomen, as the patient was very weak. The patient improved after the removal of the fluid, which did not reaccumulate in any considerable quantity. The liver was found diminished in size, and from this and other circumstances, the diagnosis was cirrhosis.

*June* 28. A specimen of blood was taken from the arm for examination. She left the hospital July 6, and was confined to the bed till within a few days of her discharge.

*Analysis of the blood for cholesterine.*—The blood presented nothing peculiar in its appearance. A quantitative analysis was made for cholesterine, with the following results:—

| | |
|---|---|
| Quantity of blood, . . . . . . . | 117.193 grains. |
| " " cholesterine, . . . . . . | 0.108 " |
| Proportion of cholesterine per 1,000 parts of blood, | 0.922 " |

Case IV. *Cirrhosis with ascites, and slight constitutional disturbance.*—Thomas Hughes, æt. about 33, brewer, presented himself at the Long Island College Hospital, July 1, 1862, with Dr. Dugan, of Williamsburg. He confesses to have been in the habit of drinking more or less spirit daily for the past ten years. The abdomen began to swell about eighteen months ago. The ascites was preceded by hæmatemesis, when he vomited an abundance of black, clotted blood. The belly immediately began to swell, and enlarged rapidly. He took hydragogues under the direction of a physician, and the dropsy disappeared, but returned whenever the medicines were discontinued. Œdema of the lower limbs occurred soon after the ascites commenced. He has had recurrence of hæmatemesis twice since the first attack.

He was first tapped two or three months after the affection occurred, and has been tapped about thirty times since. Was tapped last on the 27th ult. *He is tapped and goes out the next day. He thinks nothing of it, and is always for the time relieved.* He has continued to drink beer daily and some spirit. After tapping his appetite is good, and food occasions no inconvenience. When he is full, food occasions a distressing distention, so that he does not eat freely.

Urine is scanty when the abdomen is full, and free, after tapping.

There is no pain in the belly, or elsewhere. He is about all day, but is not engaged in business. He says he is not very feeble. He presents a notably anæmic aspect.

The abdomen is now moderately full, (July 1.) Superficial veins of abdomen much enlarged. Heart appears not enlarged; a feeble systolic murmur over the body of the organ.

Several months before the ascites began, he got into a fracas, and was badly beaten. He was not laid up, but says he did not feel well afterward, and is disposed to attribute his disease thereto.

Advised to continue to tap when the abdomen refills, with tonics, hygienic measures, and abstinence from spirit, continuing the use of ale moderately. (*Private records of Dr. Flint.*)

*July* 1. A specimen of blood was taken from the arm for examination.

*Analysis of the blood for cholesterine.*—The blood was treated in the usual way, and a quantitative analysis made for cholesterine, with the following results:—

| | |
|---|---|
| Quantity of blood, . . . . . . . | 251.567 grains. |
| " " cholesterine, . . . . . . | 0.062 " |
| Proportion of cholesterine to 1,000 parts of blood, | 0.246 " |

The following table shows the comparative quantity of cholesterine in these specimens, and in the three specimens of healthy blood:—

| Healthy Blood. | Cholesterine per 1,000 pts. | Blood of Cirrhosis. | Cholesterine per 1,000 pts. |
|---|---|---|---|
| Male, æt. 35, . . | 0.445 | Case III. Cirrhosis, (severe) | 0.922 |
| " " 22, . . | 0.658 | Case IV. Cirrhosis, (mild) | 0.246 |
| " " 24, . . | 0.751 | | |

| | |
|---|---|
| Case III. Cirrhosis (severe). Percentage of increase in cholesterine over minimum of healthy blood, . | 107.190 |
| Ditto over maximum, . . . . . | 22.769 |
| Case IV. Cirrhosis (mild). Percentage of decrease in cholesterine below minimum of healthy blood, . | 42.469 |

These two cases present a very striking contrast; and the chemical examination of the blood has shown as marked a difference in the quantity of cholesterine, as in the gravity of the attendant symptoms. It teaches, however, an important lesson. *We do not always have an accumulation of cholesterine in the blood when the structure of the liver is altered; it being requisite that this alteration should involve enough of the organ to interfere with the elimination of this substance.* The quantity may even fall below the natural standard, in a patient who is rendered anæmic by the consequences of a cirrhosis, which is not sufficient to induce cholesteremia. The process of nutrition being thereby diminished in activity, the production of this substance, by destructive assimilation, is necessarily diminished. The cholesteremia may be slight and transient; for the causes which produce it may be to a certain extent, temporary. In Case III. we have the patient confined to the bed, suffering acute pain over the region of the liver, in all probability due to a slight degree of inflammation. This interfered with the excretion of cholesterine, and we find its proportion in the blood increased to 22.769 per cent over the maximum, and 107.190 over the minimum.* As the patient was somewhat enfeebled by syphilis before the symptoms of disease of the liver made their appearance, it is probable that the quantity of cholesterine in the blood did not mount up to the highest standard in health. At all events, there was a notable increase even over the maximum quantity. Case IV. is not less instructive. Here we have a patient who has had cirrhosis of the liver, with ascites, for eighteen months, and has been tapped upwards of thirty times. He apparently has suffered from nothing more than the mechanical effects of the liquid, which has interfered at times with digestion, and rendered him anæmic. He is tapped, and immediately relieved, going out the next day. We do not seem to have any interference with the functions of the liver, as far as the symptoms are concerned, other than the mechanical obstruction to the circulation, and the case, in its symptoms, resembles one of those cases of ovarian dropsy, where we have the patient carrying about an immense quantity of water, but suffering only from this circumstance, and relieved temporarily when the water is removed. Considering the state of the patient, we should not be surprised to find the cholesterine of the blood not increased, but diminished in quantity; and we may, I think, come to the conclusion from the symptoms, as well as the analysis of the blood, that though the liver was affected sufficiently to produce obstruction of the circulation, there was not sufficient disease to produce cholesteremia.

It is evident that much more extended observations are necessary in order to establish the clinical relations of cholesteremia without jaundice; but the case of Mary Perkins shows that this condition does exist, while the case of Thomas Hughes shows that it does not follow structural change in the liver, unless the lesion be extensive. The fact that we may have poisoning of the blood by the retention of a biliary matter, *without discoloration of the skin*, is exceeding important; and of this there seems to me to be no doubt. When we have a patient who has structural disease of the liver, and presents symptoms of blood-poisoning, he is suffering under *cholesteremia*, though there be no icterus. The cholesteremia may vary in degree from the mildness which characterized Case III., in which it was, perhaps, temporary,† to the grave condition mentioned

* Unfortunately the character of the stools was not noted.

† The patient having gone out of the hospital, it was impossible to settle this point experimentally, by Frerichs, characterized by noisy delirium and coma, and announcing a speedy fatal termination. When we add to these conditions the cases of what is ordinarily called biliousness, attended with drowsiness, an indefinite feeling of *malaise*, constipation, etc., (and all this relieved by a simple mercurial purge, which is said to promote the secretion of the liver), cannot we hope that some light will be shed on their pathology by a knowledge that there is a condition called cholesteremia? As yet there is but speculation; but the discovery of the important function of cholesterine opens an almost boundless field of inquiry in this direction; and ere long the physician may talk of "biliousness," and "liver complaint," with some definite ideas of their pathology.

The following table gives the results of the quantitative analyses for cholesterine, which have been referred to in this article:—

*Table of Quantitative Analyses for Chlosterine.*

| | | Quantity examined. | Cholesterine per 1,000 pts. |
|---|---|---|---|
| | | grains. | |
| Human blood from the arm. | Healthy male æt. 35 | 312.083 | 0.445 |
| " " " | " " æt. 22 | 187.843 | 0.658 |
| " " " | " " æt. 24 | 102.680 | 0.751 |
| " " " | Simple jaundice, | 212.428 | 0.508 |
| " " " | Cholesterema with jaundice | 50.776 | 1.850 |
| " " " | Cirrhosis (grave) | 117.193 | 0.922 |
| " " " | " (mild) | 251.567 | 0.246 |
| " " " | Hemiplegia— | | |
| | Case I. Paralyzed side | 55.458 | — |
| | Sound side | 128.407 | 0.481 |
| | Case II. Paralyzed side | 18.381 | — |
| | Sound side | 66.396 | 0.808 |
| | Case III. Paralyzed side | 21.824 | — |
| | Sound side | 52.261 | 0.579 |
| Blood from carotid | Dog experiment | 179.462 | 0.774 |
| " " internal jugular | Dog experiment | 134.780 | 0.801 |
| " " femoral vein | Dog experiment | 133.886 | 0.806 |
| " " carotid | Dog experiment | 140.847 | 0.768 |
| " " internal jugular | Dog experiment | 97.811 | 0.947 |
| " " carotid | Dog experiment | 143.625 | 0.967 |
| " " internal jugular | Dog experiment | 29.956 | 1.545 |
| " " femoral vein | Dog experiment | 45.035 | 1.028 |
| " " carotid | Dog experiment | 159.537 | 1.257 |
| " " portal vein, | Dog experiment | 168.257 | 1.009 |
| " " hepatic vein | Dog experiment | 79.848 | 0.964 |
| Human brain (subject killed instantly) | | 159.753 | 7.729 |
| " Case II., (killed instantly) | | 150.881 | 11.456 |
| Human bile (specimen from Case II.) | | 224.588 | 0.618 |
| Crystalline lens (4 lenses from the ox) | | 135.020 | 0.907 |
| Meconium | | 170.541 | 6.245 |

Conclusions.—The observations contained in the preceding article seem to the writer to justify the following conclusions:—

1. Cholesterine exists in the bile, the blood, the nervous matter, the crystalline lens, and the meconium, but does not exist in the feces in ordinary conditions. The quantity of cholesterine in the blood of the arm is from five to eight times more than the ordinary estimate.

2. Cholesterine is formed, in great part if not entirely, in the substance of the nervous matter, where it exists in great abundance, from which it is taken up by the blood, and constitutes one of the most important of the effete or excrementitious products of the body. Its formation is constant, it always existing in the nervous matter and the circulating fluid.

3. Cholesterine is separated from the blood by the liver, appears as a constant element of the bile, and is discharged into the alimentary canal. The history of this substance, in the circulating fluid and in the bile, mark it as a product destined to be gotten rid of by the system, or an excretion. It pre-exists in the blood, subserves no useful purpose in the economy, is separated by the liver and not manufactured there, and, if this separation be interfered with, accumulates in the system, producing blood-poisoning.

4. The bile has two separate and distinct functions dependent on the presence of two elements of an entirely different character. It has a function connected with nutrition. This is dependent on the presence of the glyco-cholate and tauro-cholate of soda, which do not pre-exist in the blood, subserve a useful purpose in the economy, and are not discharged from it, are manufactured in the liver and peculiar to the bile, do not accumulate in the blood when the function of the liver is interfered with, and are, in short, the products of *secretion.* But it has another function connected with depuration, which is dependent on the presence of the cholesterine, which is an *excretion.* The flow of the bile is remittent, being much increased during the digestive act, but produced during the intervals of digestion, for the purpose of separating the cholesterine from the blood which is constantly receiving it.

5. The ordinary normal feces do not contain cholesterine, but contain *stercorine* (formerly called seroline, from its being supposed to exist only in the serum of the blood), produced by a transformation of the cholesterine of the bile during the digestive act.

6. The change of cholesterine into stercorine does not take place when digestion is arrested, or before this process commences; consequently, stercorine is not found in the meconium, or in the feces of hibernating animals during their torpid condition. These matters contain cholesterine in large abundance, which also sometimes appears in the feces of animals after a prolonged fast. Stercorine is the form in which cholesterine is discharged from the body.

7. The difference between the two varieties of jaundice with which we are familiar, the one characterized only by yellowness of the skin, and comparatively innocuous, while the other is attended with very grave symptoms, and is almost invariably fatal, is dependent upon the obstruction of the bile in the one case, and its suppression in the other. In the first instance, the bile is confined in the excretory passages, and its coloring matter is absorbed, while in the other, the cholesterine is retained in the blood, and acts as a poison.

8. There is a condition of the blood dependent upon the accumulation of cholesterine which I have called *Cholesteremia.* This only occurs when there is structural change in the liver, which incapacitates it from performing its excretory functions. It is characterized by symptoms of a grave character, referable to the brain, and dependent upon the poisonous effects of the retained cholesterine on this organ. It occurs with or without jaundice.

9. Cholesteremia does not occur in every instance of structural disease of the liver. Enough of the liver must be destroyed to prevent the due elimination of the cholesterine. In cases in which the organ is but moderately affected, the sound portion is capable of performing the eliminative function of the whole.

10. In cases of simple jaundice, when the feces are decolorized and the bile is entirely shut off from the intestine, stercorine is not found in the evacuations; but in cases of jaundice with cholesteremia, the stercorine may be found, though always very much diminished in quantity, showing that there is an insufficiency in the separation of the cholesterine from the blood, though its excretion is not entirely suspended. After death, but a small quantity of bile is found in the gall-bladder.

In concluding, I beg leave to express my acknowledgments to my assistant, Mr. Henry E. Paine, of Providence, R. I., one of the residents of the Bellevue Hospital, who has assisted me indefatigably in the analyses and experiments which form the basis of this paper; and to whose intelligent aid I am greatly indebted for the amount of labor which I was enabled to accomplish in a comparatively short time. This aid, with the advantages of the great hospital at Blackwell's Island, which was laid under contribution for the pathological observations, enabled me to carry on a portion of the analyses uninterruptedly for about two months.—*American Journal of the Medical Sciences.*

